# Adapting the World Health Organization iSupport Dementia program to the Indonesian socio-cultural context

**DOI:** 10.3389/fpubh.2023.1050760

**Published:** 2023-02-16

**Authors:** Yuda Turana, Kevin Kristian, Ika Suswanti, Tara Puspitarini Sani, Yvonne Suzy Handajani, Kham Tran, Tuan Anh Nguyen

**Affiliations:** ^1^School of Medicine and Health Sciences, Atma Jaya Catholic University of Indonesia, Jakarta, Indonesia; ^2^Center of Health Research, School of Medicine and Health Sciences, Atma Jaya Catholic University of Indonesia, Jakarta, Indonesia; ^3^Social Gerontology Division, National Ageing Research Institute, Melbourne, VIC, Australia; ^4^School of Health Sciences, Swinburne University of Technology, Melbourne, VIC, Australia; ^5^UniSA Clinical and Health Sciences, University of South Australia, Adelaide, SA, Australia; ^6^Health Strategy and Policy Institute, Ministry of Health, Hanoi, Vietnam

**Keywords:** cultural adaptation, dementia, Indonesia, iSupport, WHO

## Abstract

**Background:**

Providing care for people with dementia (PwD) without sufficient understanding of the condition might negatively affect the wellbeing of the caregivers, given the lengthy care and progressive nature of the disease. The iSupport for dementia developed by World Health Organization (WHO) is a self-administered training manual for caregivers of PwD, adaptable to local cultures and contexts. This manual needs translation and adaptation to produce a culturally appropriate version for use in Indonesia. This study reports the outcomes and lessons learnt from our translation and adaptation of iSupport content into Bahasa Indonesia.

**Methods:**

The original iSupport content was translated and adapted using the WHO iSupport Adaptation and Implementation Guidelines. The process included forward translation, expert panel review, backward translation, and harmonization. The adaptation process included Focus Group Discussions (FGD), involving family caregivers, professional care workers, professional psychological health experts, and Alzheimer's Indonesia representatives. The respondents were asked to express their opinions about the WHO iSupport program, which comprises five modules and 23 lessons covering well-established topics on dementia. They were also asked to suggest improvements and their personal experiences compared to the adaptations applied in the iSupport.

**Results:**

Two experts, 10 professional care workers, and eight family caregivers participated in the FGD. Overall, all participants had positive views of the iSupport material. The expert panel identified the need to reformulate definitions, recommendations, and local case studies to fine-tune the original contents to local knowledge and practices. Based on the feedback in the qualitative appraisal, several improvements regarding the language and diction, additional relevant and concrete examples, personal names and cultural habits, and customs and traditions were addressed.

**Conclusions:**

The translation and adaptation of the iSupport into the Indonesian context have shown some changes needed to make the iSupport content culturally and linguistically appropriate for Indonesian end users. In addition, given the broad spectrum of dementia, various case illustrations have been added to improve the understanding of care in particular situations. Future studies are needed to evaluate the efficacy of the adapted iSupport in improving the quality of life of PwD and their caregivers.

## 1. Introduction

In the last decade, the Indonesian older population has gradually increased, from 7.6% of the whole population (~18.0 million people) in 2010, to about 10.8% or 29.3 million people in 2021 (1, 2). This indicates that Indonesia has entered the phase of population aging that is marked by increasing age-ratio independence in older populations (3).

The increase in the older population may lead to not only several health problems, which are mainly degenerative diseases, including dementia, but also a higher burden of care. Based on a systematic analysis of Indonesia's Global Burden of Disease (GBD), Alzheimer's Disease (AD) jumped from 33^rd^ in 2006 to 24^th^ in 2016 as the leading cause of Disability-Adjusted Life Years (DALYs) (4, 5).

Caregivers (family and professional caregivers) have a crucial role, especially for the older people who have experienced a functional decline. In simple terms, a caregiver is a person who tends to the needs or concerns of a person with short- or long-term limitations due to illness, injury or disability. The term “family caregiver” describes individuals who care for members of their family of origin, but also refers to those who care for their family of choice. This could be members of their congregation, neighbors or close friends (6). A professional caregiver is hired to provide care for a care recipient. These caregivers can provide medical or non-medical care in the home or a facility (7).

Regarding the provision of care for people living with dementia (PwD), problems related to poor awareness and understanding of dementia often lead to stigmatization and delay in diagnosis and treatment (8). Several studies in Indonesia indicated a lack of knowledge about dementia. Research by Suriastini et al. showed that only 9.9% of caregivers of older people in Yogyakarta knew that memory loss is a symptom of Dementia (9). Other studies also revealed that more than 67% of the study respondents had poor knowledge about dementia (10). A poor understanding of dementia and the burden of care among family caregivers is an important issue that is frequently missed. Caring for PwD is generally long-term, often resulting in a high burden on caregivers and family members. According to a study by Nasrun et al., family caregivers spent more than 10 h per day looking after PwD (11). In comparison, the World Health Organization (WHO) estimated 5 h on average per day (8). This, along with physical, emotional, and financial pressures, may induce great stress on family members and caregivers, suggesting the need for social support.

As caregivers of PwD often deal with many challenges, failure to adapt to caregiving roles may disrupt them physically and mentally. A study by Putri et al. showed that one in four caregivers of PwD (24.8%) showed symptoms of depression (12). This study indicated that the quality of life of the PwD is closely related to the quality of care that caregivers provide.

Considering the cost and sustainability aspects, non-pharmacological approaches are needed to support caregivers in improving their mental health. The development of accessible, acceptable, and practical training, as well as supportive interventions for family caregivers of PwD, is emphasized as a strategic priority in the global action plan on the public health response to Dementia 2017–2025. To this end, WHO has developed *iSupport for Dementia*, a generic version of an *online skill training* and *evidence-based* program for family caregivers of PwD that can be culturally and linguistically adapted for use in different local contexts (13). The study shows that the online iSupport program is feasible for engaging carers in self-learning. The findings also confirm that the program can embed information on dementia care services throughout learning modules for carers to access instantly when needed. In addition, in recent years, there has been an increase in the use of gadgets and the internet in Indonesia. This became even more evident during the COVID pandemic, which limited direct physical meetings and online learning (14). The survey in Indonesia showed an increase in internet using by 64.8% in 2018, and 77.02% in 2021–2022 (15).

Though Indonesia is a large country with 1,340 ethnic groups and 2,500 local languages, it is still lacking on evidence-based online training and support programs for informal dementia caregivers. Therefore, we aim to adapt iSupport to fit local Indonesian culture and context. The cultural adaptation of an intervention program is systematically adjusting its elements to the language, culture, and context to match a particular group's cultural patterns, meanings, and values (16–18). This paper describes the development of the culturally adapted version of the WHO iSupport program for family caregivers of PwD within the Indonesian context, through 5 stages including: assessment, content translation, cultural adaptation, expert panel appraisal and fidelity check, as part of the e-DiVA project to develop an iSupport Virtual Assistant to support family caregivers of PwD in Australia, Indonesia, New Zealand and Vietnam (19).

## 2. Materials and methods

### 2.1. The iSupport Dementia

**Figure 1:**
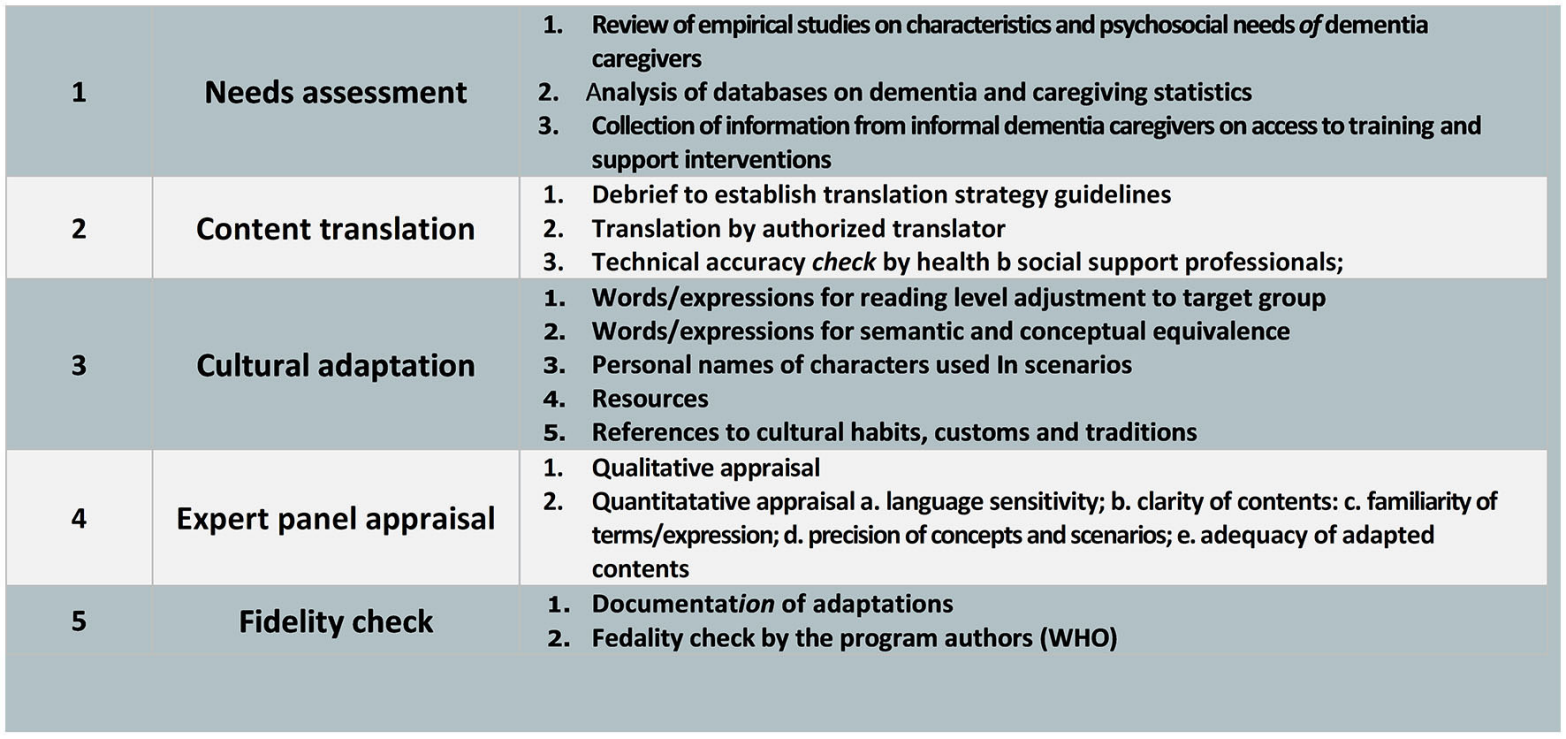
Methodological approach to adapt an evidence-based online intervention for informal dementia caregivers (iSupport): A five-step procedure (21).

**Figure 2:**
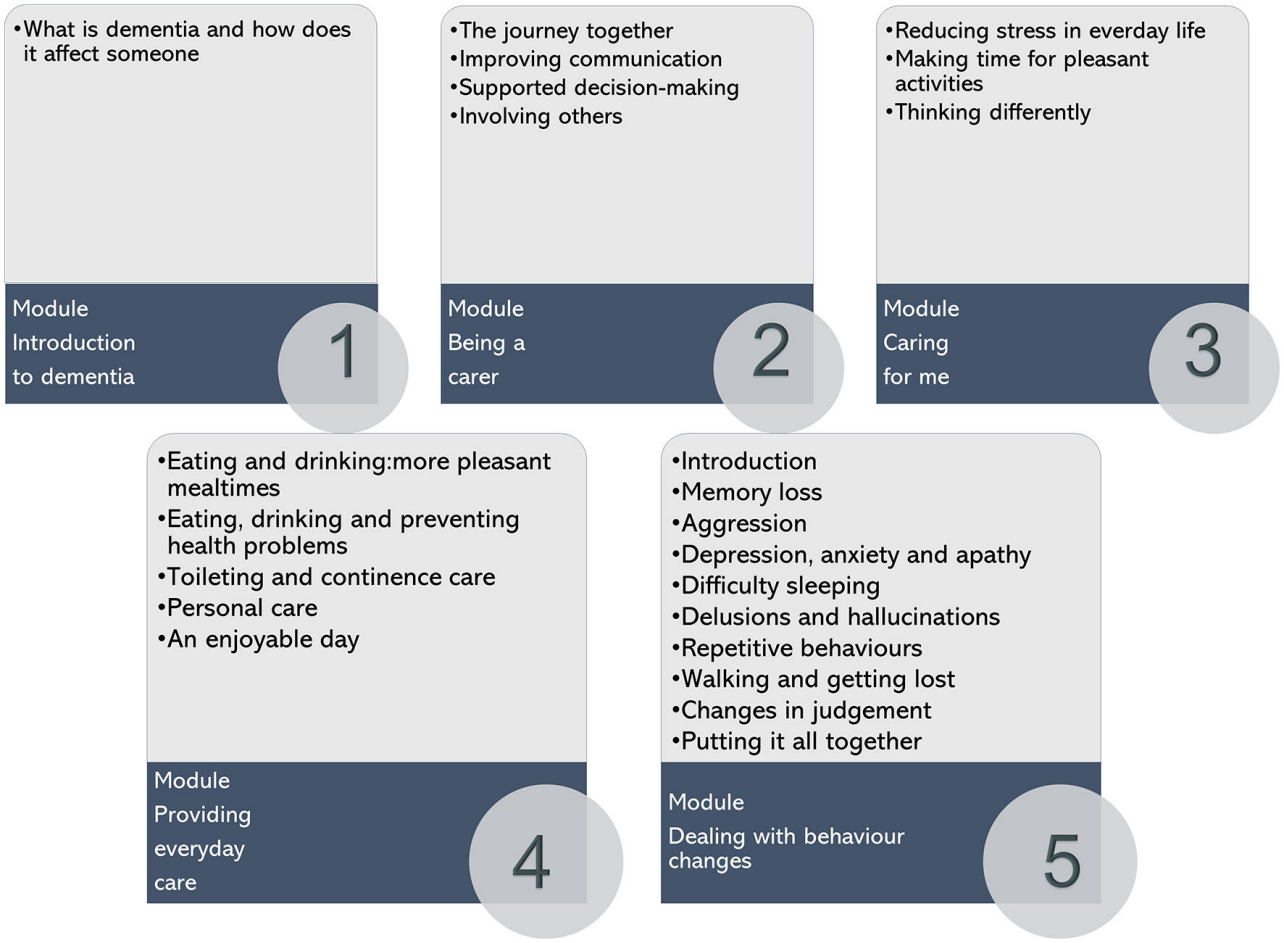
Overview of the iSupport online self-help training and support programme: Structure and contents (13).

The iSupport is an e-learning or online self-help program designed to provide education, skills training, and social support to family caregivers of PwD. E-learning is an approach to teaching and learning, representing all access to training, communication and interaction, and that facilitates the adoption of new ways of understanding and developing learning (20). The e-learning program comprises five modules, containing 23 lessons that cover well-established topics on dementia and caregiver support (cf. Figure 1). The iSupport manual includes five modules and accompanying exercises, namely: (i) introduction to dementia; (ii) being a caregiver; (iii) caring for me; (iv) providing everyday care; and (v) dealing with behavior changes (Figure 2). The modules and exercises reflect those included in the online version of iSupport. The iSupport manual can be printed and used offline, allowing for a broad reach of the programme, particularly in regions with low internet bandwidth and connectivity.

### 2.2. Translation and adaptation methods

#### 2.2.1. Need assessment

This stage is the initial process of identifying study needs, which consists of understanding the study concept, searching for literature, and gathering information related to the characteristics of caregivers who will participate in the form of a checklist of names obtained from the Alzheimer's Indonesia.

#### 2.2.2. Content translation and technical accuracy check

The text of the iSupport in the generic version was translated from English into Bahasa Indonesia. The Translation was conducted by an authorized translator, following the WHO iSupport Adaptation and Implementation Guide (22) and the WHO Process of Translation and Adaptation of Instruments (23), which focus on cross-cultural and conceptual equivalence. The process included forward Translation, review by an expert panel, backward Translation, and harmonization to address discrepancies to produce a satisfactory version. The first version of the translated iSupport went through a technical accuracy check by an expert panel of healthcare professionals in dementia care, including two psychologists and ten geriatric nurses.

The translation process was carried out following an interpretative-communicative method (24), using translation techniques that are needed according to the discursive and contextual characteristics of the source text unit. The result shows that the translator has used local and global adaptation features to better describe the program contents without changing the meaning. In translating the text, the translator did not only retain its meaning but also considered the effectiveness and beauty of the discourse.

The translation process was preceded by a debrief between the principal investigator and the translator to establish translation strategy guidelines, including adopting international standards on the language that describes people with dementia. Afterwards, the translators were allowed to raise questions regarding the technical meaning or context that attracted the project's principal investigator's attention and propose different translation options open for discussion. A team of three researchers went over the results of the translation process performed by professional translators word for word. If there are divergent opinions among researchers, a forum is held to reach a consensus with all researchers based on the opinions of the majority.

#### 2.2.3. Cultural adaptation

After checking the content translation and technical accuracy, the local adaptation of the iSupport was organized with guidance from adaptation directives made available by WHO. These directives covered the adaptation of contents *per se* by providing a list of text contents which might require local adaptation and advice on general approaches to the adaptation work.

First, the research team conducted a preliminary adaptation of contents considering the orientations provided in WHO's guide (adaptation phase 1). The elements subjected to cultural adaptation included: (1) Words and expressions for reading-purpose adjustments to the targeted group; (2) Words and expressions for semantic and conceptual equivalence; equality in the suitability of word placement with the meaning to be conveyed, for example: disease–illness; essential-important; social class-social status etc. (3) Personal names of the characters that are used in the iSupport scenarios; (4) Resources (e.g., information materials, services available, technologies and products/services available), and (5) References to cultural habits, customs, and traditions (e.g., leisure activities, food, customary practices). Neither of these changes the core aspects of the intervention, alterations to scenarios, nor answer options of the interactive exercises was proposed. The researcher did not change any aspect of the iSupport module, including scenarios. The proposed adaptations were regarded on the cultural knowledge of the research team, who are fully native Indonesians, based on the observable characteristics, experiences, norms, values, as well as behavioral patterns and beliefs of the Indonesian populations in general. Most of the researchers are native Indonesian speakers, so we have experience in identifying the characters and values that develop in Indonesian society, so this becomes our reference in adopting. Consequently, with various kinds of culture that have developed in Indonesia, we can only assess the general picture but do not apply to local indigenous cultures in certain areas.

##### 2.2.3.1. Focus group discussions

Three FGDs were held in Bahasa Indonesia to discuss each module. FGD participants included family caregivers and professional caregivers, in which the FGD was conducted separately.

###### 2.2.3.1.1. Recruitment participant

FGD participants were recruited based on the information contained in the list of caregivers for the Alzheimer's Indonesia. There were 18 participants with details of 8 family caregivers, 10 professional caregivers. The first FGD was conducted on family caregivers and the second FGD was conducted on professional caregivers.

Within each group, participants will be recruited using a purposive sampling technique where the inclusion criteria for family caregivers are family members living at home and caring for PwD for 2 years or more. In the family caregiver group, the inclusion criteria were people who had professionals working with PwD for 2 years or more.

###### 2.2.3.1.2. Procedure of FGD

Participants will be given at least 2 weeks to review the hard copy of the adapted iSupport individually and asked to write down their opinions about content that needs attention before the FGD. In the FGD, participants will discuss the lessons of iSupport in depth with items that come up and seem essential to the group members. At the time of implementation, the FGD was guided by one facilitator. We used a checklist of questions as a guide for the FGD. Each participant was given 10–20 min to make suggestions. If there were points that have not been conveyed during the FGD, participants could send an email or other online media afterwards. The facilitator will direct the course of the FGD by providing questions and feedback to the participants, and one person will record the FGD input points.

The 2-h FGD was arranged by an online meeting. The activities in each FGD were recorded. The recording results were transcribed for later review by the research team and analyzed using thematic analysis to see any significant and minor feedback. The thematic analysis was carried out covering several stages including familiarizing the data, generating initial codes, searching for themes, reviewing themes, defining and naming themes, producing the repot (21).

#### 2.2.4. Expert panel appraisal

The appraisal was done both qualitatively and quantitatively through identification of sensitive words, clarity content, familiarity of terms, expression, precision concept and scenario by multi background expert, involving psychological experts and representatives of the Alzheimer's Indonesia.

#### 2.2.5. Fidelity check

The final translated product was then sent to WHO for further quality assurance. All changes regarding the texts or illustrations were recorded on the WHO iSupport adaptation forms to be submitted to the WHO for approval.

### 2.3. Ethical statement

This study has obtained ethical clearance with letter number: 14/02/KEP-FKIKUAJ/2021 from the School of Medicine and Health Sciences Atma Jaya Catholic University of Indonesia. This study has obtained the consent of the participants.

## 3. Results

### 3.1. The iSupport program content

Depending on the stage of dementia, the examples in the iSupport program differ from those our caregiver participants have encountered. Self-checks should be created, if possible, so that readers can determine which stage of dementia they are dealing with and which areas require special attention.

The severity of dementia is related to the approach that should be taken; therefore, the behavioral interventions that should be carried out are different at each stage. In the iSupport program, the behavioral changes that may occur at specific stages were presented, along with the role of caregivers in each of these stages. Unfortunately, it was described with the relatively uncommon Indonesian metaphor of colored balloons representing emotions. In light of this, it would have been more appropriate for the Indonesian context to provide examples in the form of images when addressing this issue.

Regarding hotline and contact person, the methods outlined in the source text should be replaced with those commonly employed in Indonesia. For instance, the source text highlighted the necessity of contacting the clinics and making doctor's appointments. In the context of Indonesia, a local cadre is the closest point of contact with the local population and should replace a clinic.

In the original iSupport program, the majority of tasks for caregivers were provided in the form of a blank table, so caregivers could fill it in as necessary. To guide caregivers in the Indonesian context, it is preferable, however, to provide an example at the top of the table. Also, simple matters may become complicated if caregivers must read for an extended period of time. Given Indonesians' propensity for not reading, it is preferable to include more illustrations or visuals in the translated and adapted version of the program.

### 3.2. Translation and adaptation result

#### 3.2.1. Need assessment

From the list of caregivers at the Alzheimer's Indonesia, the names of 8 family caregivers and 12 professional caregivers were obtained. Table 1 contains the characteristics of the respondents.

**Table 1:** Socio demography characteristics of FGD participant.

**Family caregiver**	***N* (%)**
**Gender**	
Male	1 (12.5)
Female	7 (87.5)
Age [Mean (SD)]	45.3 (14.8)
**Education**	
Senior high school	1 (12.5)
Undergraduate	7 (87.5)
Postgraduate	0 (0.0)
**Marital status**	
Single	4 (50.0)
Married	4 (50.0)
**Relationship with PwD**	
Parents	5 (62.5)
Spouse	2 (25.0)
Relatives	1 (12.5)
Years of caring [Mean (SD)]	7.1 (2.9)
**Professional caregiver**	***N*** **(%)**
**Gender**	
Male	3 (25.0)
Female	9 (75.0)
Age	45.0 (11.6)
**Education**	
Senior high school	0 (0.0)
Undergraduate	2 (16.7)
Postgraduate	10 (83.3)
**Marital status**
Single	2 (16.7)
Married	10 (83.3)
Years in the field of dementia care [Mean(SD)]	12.7 (6.3)

The mean age of family caregivers ranged was 45.3 (SD:14.8) years, while the age of professional caregivers was 45.0 (11.6) years. Both groups were predominantly female. All participants had at least a primary level of education (consisting of a minimum of 6-year study). All respondents use Bahasa Indonesia in their daily life.

The study's main focus was various inputs related to diction and language, content, and recommendations. Several improvements were identified in the Translation and adaptation of WHO iSupport program to the local Indonesian culture. Moreover, linking the word choice to the glossary would help users better understand medical and technical terms. In addition, it is also helpful to mention the local cultures in the program as part of the content to make it more relevant. Furthermore, providing examples in the task session is necessary since several formal words in Bahasa Indonesia are rarely used daily.

#### 3.2.2. Content translation and technical accuracy check

During the translation and adaptation process, there were several instances in which, despite rigorous and independent Translation conducted, specific items did not make sense to the participants and needed additional clarifications. Here we provide examples of such instances and subsequent changes to the module, as follows: Table 2.

**Table 2:** Translation backward and forward, and technical accuracy.

**Original iSupport**	**Backwards TL**	**Forwards TL**	**Notes**
**Module 1**			
Why is this lesson important? Dementia is a disease that can be overwhelming for the person with dementia, but also for you as a carer.	Why is this lesson important? Dementia is an illness that could affect both the patient and their carers greatly.	Mengapa pelajaran ini penting? Demensia merupakan suatu penyakit yang dapat berdampak besar bagi orang dengan demensia, dan Anda sebagai pengasuhnya.	
**Module 2**			
**Why is this lesson important?** Dealing with dementia is a journey you will take together, because it changes the daily life of the person living with dementia and the carer.	**Why is this lesson important?** Facing with dementia is a journey that you can experience together, as this will change the day- to-day life of the person with dementia as well as their carers.	**Mengapa pelajaran ini penting?** Berhadapan dengan demensia adalah suatu perjalanan hidup yang akan Anda alami bersama, karena hal ini akan mengubah kehidupan sehari-hari dari orang dengan demensia beserta pengasuhnya.	
**Module 3**			
2. Different ways to relax: • Basic breathing; • Mindful breathing; • Neck movements; • Number counting; • Imagery; • Total stretching; • Muscle relaxation.	2. How to relax. • The basics of breathing • Mindfulness • Neck movement • Counting numbers • Imaging • Total stretching • Muscle relaxation	2. Berbagai cara untuk rileks. • Dasar pernapasan; • Bernapas dengan kesadaran *(mindful)*; • Pergerakan leher; • Menghitung angka; • Pencitraan; • Peregangan total; • Relaksasi otot.	2. Berbagai cara untuk rileks. • Dasar pernapasan; • Bernapas dengan penuh kesadaran *(mindful)*; • Pergerakan leher; • Menghitung angka; • Imajinasi; • Membayangkan; • Peregangan total; • Relaksasi otot.
**Module 4**			
**Module 4: Providing** **everyday care** • Lesson 1. Eating and drinking–more pleasant mealtimes. • Lesson 2. Eating and drinking–preventing health problems. • Lesson 3. Toileting and continence care. • Lesson 4. Personal care. • Lesson 5. An enjoyable day.	**Module 4: Providing everyday care** • Lesson 1: Eating and drinking–eating time that is more enjoyable. Lesson 2: Eating, drinking and preventing eating problems. • Lesson 3: Continence care and toileting. • Lesson 4: Personal care. • Lesson 5: A fun day.	**Modul 4: Memberikan perawatan setiap hari** • Pelajaran 1: Makan dan minum–waktu makan yang lebih menyenangkan. • Pelajaran 2: Makan, minum, dan mencegah masalah Kesehatan. • Pelajaran 3: Perawatan kontinensia dan toileting. • Pelajaran 4: Perawatan pribadi. • Pelajaran 5: Hari yang menyenangkan.	
**Module 5**			
How will this lesson help me? This lesson helps to improve your skills to prevent and cope with behavior changes.	How can this lesson help me? This lesson helps you improve your skills to prevent and deal with behavioral change.	Bagaimana pelajaran ini dapat membantu saya? Pelajaran ini membantu Anda memperbaiki keterampilan Anda untuk mencegah dan berhadapan dengan perubahan perilaku.	Bagaimana pelajaran ini dapat membantu saya? Pelajaran ini membantu Anda meningkatkan keterampilan Anda untuk mencegah dan mengatasi perubahan perilaku.

#### 3.2.3. Cultural adaptation

In the FGD with the caregivers: The respondents understood the modules very well, although several sentences needed to be paraphrased to achieve greater clarity (see Table 3). The length of each lesson was more of an issue for family caregivers but not for professional caregivers. In discussions with the family caregivers, several topics would not be able to represent the actual conditions in the daily care experienced by the caregivers, as it also depends on the severity of the PwD. For example, in module 5, there is a topic on how PwD cares for behavior disorders in PwD. This situation may not benefit caregivers who provide care for PwD in the early phase because behavior change in the early stages is not apparent. In discussion with a panel of experts consisting of psychologists and Alzheimer's Indonesia representatives, the WHO iSupport program was thought to be helpful but needed several adaptations to fit the Indonesian culture. It is also believed that this program does not accurately depict the conditions of the various phases of dementia. Therefore, several additional case examples are required to encompass the disorder's diversity.

**Table 3:** Adjusting for cultural adaptation.

**Categorical**	**Item change**
The graphics and illustrations	• Some cartoons and colors that depict emotional situations needed further adjustments to the Indonesian culture. For example, a cartoon balloon may be best drawn with a human image. • The program is better to be reproduced with more illustrations. • There is an illustrated story about PwD, who has memory loss. It would be more interesting if there were illustrations about it. Events need to be adapted to culture, for example, on page 13 of the Indonesian-translated version of the book where PwD has difficulty in shopping. • Some cartoons and colors that depict emotional situations needed further adjustments to the Indonesian culture. For example, a cartoon balloon may be best drawn with a human image.
Terms and expressions	• Some unfamiliar words, such as continence, toileting, and delusion, then these words were changed to the following terms: continence (gangguan kontrol buang air), toileting (kemandirian bertoilet), delusion (khayalan). • The translation language is good, but it is better to translate it using everyday practical language. • Some examples of behavior changes, such as hallucinations, were not considered as deep enough and thought to be inappropriate to what truly is experienced by the respondents. This program was thought to be too superficial and needed to be strengthened on the aspects of behavior changes. • The symptoms of dementia are part of a broader spectrum. Hence, at the beginning of the program, it is necessary to self-check in which stage PwD is the caregivers are dealing with and what is essential to pay more attention to. • Some medical terms that already had been adapted to standard Indonesian, namely incontinence, were still considered too medical. However, it is known to be a settled terminology in Bahasa Indonesia, which later had to be re-adapted and explained with a written explanation of not being able to hold urination or bedwetting, thus it is essential to add a glossary for complex languages.
Content	• Material regarding ADL should be discussed in the module. Since in the late stage of dementia, almost all PwD have impairment in ADL. • This manual contains relaxation exercises. Instructions must be more structured and practical to make this self-support program easier.

##### 3.2.3.1. Word expression for semantic and reading level adjustment

The caregiver is already a term in Bahasa Indonesia which means a person who provides care or companionship. However, “companion” is used more often than “caregiver.” Additionally, the word “companion” also gives more positive value even though the word companion is not entirely similar to a caregiver in English.

In English and Western culture, the word dementia seems familiar among laypeople, albeit it is a medical term. However, this term is a medical term in the Indonesian context, and is seldom used as a daily language, so laypeople do not know it, nor is it widely comprehended. Moreover, it is also often translated as “senile” in the Indonesian context, although the term “senile” refers to a “memory” disorder only that does not quite represent the whole condition. Besides, the term “senile” is also used as a tagline to increase awareness of dementia in the community.

Moreover, several words, such as mindful breathing, were also translated as “breathing with complete awareness.” There is no Indonesian equivalent for the word “mindful,” but a different explanation is required to make the uncommon word more understandable. Some activities, such as crossword puzzle games, are rarely performed by older people in Indonesia, particularly those with a lower educational status, who make up the majority of the older population in Indonesia. Therefore, it is necessary to alternate various examples of activities, such as social gatherings and praying, in order to create a more pertinent meaning among the older Indonesian population.

##### 3.2.3.2. Personal names

Personal names are deeply rooted in the language speakers' culture. Translating and adapting personal names are challenging and require sensitive decision-making, especially without clear translation guidelines for these contents. The research team's knowledge of cultural references and figurative use of language was a crucial element in this process. As this knowledge is eminently subjective, different procedures could be and were applied in the process of translating and adapting personal names. Considering that personal names may be influenced by the characteristics of each local culture in Indonesia, the alterations in personal names according to Indonesian were carried out by the research team's FGD, who agreed to include names that fit genders and cultures in Indonesia. All names were changed to names with Indonesian cultures, such as female names: Aminah, Sumini, Lina, and Parmi or male names such as Fauzan, Toni, Budi, and Anto.

##### 3.2.3.3. Cultural habits, customs, and traditions

A significant part of the adaptation work concerned the cultural equivalence of the Indonesian version, i.e., in adapting the original contents to capture the experience of daily life in the local culture. Those contents were adapted based on the cultural knowledge of the research team, and for specific topics, empirical studies carried out with the Indonesian population were also conducted. The need to find equivalents for cultural experiences, namely habits, customs, and traditions, was identified for several content categories, including leisure activities, food, daily routines, customary practices, regular expressions, and religious practices.

There are differences in Indonesian culture regarding greeting and complimenting. Instead of “how was your day?”, which confuses most people, it was replaced by “How are you doing today?” to become more straightforward. Likewise, the latter, if someone is not related close enough, a compliment would be inappropriate. The source text also suggests affirmations like “they look nice.” Therefore, it was best replaced by “the dress suits you.” The reference to cultural habits and daily routines are also required in some cases; for example, while explaining to someone, the word “you” may be used by everybody in the Western world. However, for Indonesian, the word “you” for someone older would be inappropriate as a matter of politeness. In this case, the word 'you' should be expressed as “Sir or Madam” as older people have a higher hierarchy in the social norms.

Furthermore, regarding the bathing situation, the source text that refers to a bath or a shower only partially fits Indonesian culture. Also, a shower is not a standard tool that some people in Indonesia use. Therefore, it was replaced by a shower or a water dipper'. Instead, a water dipper is a more suitable terminology. In addition, references to religious groups, practices, or habits were also adapted (e.g., resorting to a faith community for support, saying prayers before going to sleep, praying or meditating with people with dementia).

In terms of leisure time or hobbies, activities such as “Watch birds or animals,” “Watch the clouds or explore nature,” or “Play a musical instrument” (as stated in the source text) are not typical leisure activities performed by middle-aged Indonesian or older adults. Therefore, those were replaced with more popular and culturally practiced activities such as “Go out to eat,” “Watch TV,” or “Shop for yourselves or others” (as stated in the adapted text).

Several conversations were deemed difficult to comprehend, difficult to catch, and too stiff. Therefore, it was suggested that conversations utilize the informal language typically used by older people. The use of unfamiliar medical terms or language should be accompanied by an explanation, the use of a glossary, or, if possible, a substitute word that is more appropriate for lay audiences.

#### 3.2.4. Expert panel appraisal

A panel of cross-disciplinary health psychologists, geriatric nurses, and caregivers reviewed the translated and adapted versions of iSupport independently. The executive director of the Alzheimer's Indonesia was a member of the expert panel. Additionally, other members of the expert panel are health psychology researchers.

Most of the comments and suggestions that emerged from the qualitative appraisal were also re-discussed with the native researchers involved in the preparation and Translation of iSupport adaptation. If there are sentences / words that are similar, then the appropriate word selection is based on a consensus of at least 3 researchers (most opinion). Consensus is needed, especially when modifications and replacement of several words by their synonyms, minor sentences reformulations that did not alter in any way the meaning of the text and that were related to stylistic preferences or comprehensibility improvements.

#### 3.2.5. Fidelity check

At this point, the Indonesian version of the book draft is printed. All FGD participants and the previous expert panel received hardcopy drafts of the books after the researchers double-checked them. Regarding the final, printed hardcopy version, we are looking for feedback for the next 2 weeks. Enter the information sent *via* text or email.

During the fidelity check process, most of the adaptations submitted for the Language version of iSupport were deemed accurate and consistent with the available version of the program. Most of the original versions are recommended to be retained for adaptation.

All changes regarding the texts or illustrations were recorded on the WHO iSupport adaptation forms to be submitted to the WHO for approval.

## 4. Discussion

Our study shows that the majority of respondents are women, married, and with undergraduate education. In family caregivers, PwD's relationship with caregivers is mostly parents. In line with the 2020 Bappenas study showing 79% of elderly caregivers are family members (25), this condition indicates that a family-based care approach is the main approach in caring for the elderly who need long-term care, including dementia.

The lack of information about dementia care is often the main cause of poor care. Therefore, the iSupport for dementia program developed by WHO is one of the guidelines that can be used today. Various cultural adaptations and translations of the WHO iSupport in several countries, such as China, Greece, Australia, and Brazil, have been carried out (26–29). To our knowledge, this is the first study in Indonesia to translate and culturally adapt WHO iSupport for dementia.

Tales et al. in their study regarding cultural adaptation in Portugal, showed that there were 60,000 words–was translated from British English into European Portuguese. In line with our study, phrases, names of figures are part of the adaptation process that is adapted to the local culture (30). Meanwhile, Xio et al.'s study on the Chinese community in Australia shows that in the process of cultural adaptation, not only looking at the suitability of culture, content and language through thematic analysis. This study also emphasizes the exploration of the implementation of the Chinese community iSupport program in Australia (26). In line with a study from Xiao et al. in China, the involvement of family and professional caregivers in the process of cultural adaptation and Translation would inform further revisions of the content of the iSupport program to ensure that the program is compatible with Indonesian culture. Factors affecting program implementation that were identified in the study will also be considered in the program intervention phase (26).

This study as part of the e-DiVA project (19). The e-DiVA project consists of 4 phases. The first phase focuses on Translation and local adaptation of i-Support content. This phase aims to involve people who have experience in dementia care to carry out a design to develop an iSupport VA that will be used in Indonesia. The second phase aims to test the feasibility, acceptance, and possible effects of iSupport VA through the Randomize Control Trial (RCT). Phase 3 aims to strengthen and build capacity at the local and national, clinical and academic levels to support the development and evaluation of iSupport VA through the theory of change workshop activities, and the fourth phase disseminates the results of studies.

Based on studies in other countries, terminology, language and suitability with local culture are the main focus in preparing location adaptations in each country. A similar study in Brazil showed that several changes were suggested concerning terminology, language and the healthcare system in Brazil (29).

At the current stage of the translation and adaptation process, however, a number of issues are emerging, including (1) the fact that the treatment of PwD encompasses different phases of the disease course. Therefore, it is assumed that this program is too general; (2) Although numerous terms exist in Bahasa, they are rarely used in the community. Therefore, they must be replaced to conform to Indonesian culture and customs; Several of the obstacles identified in the FGDs concern the use of an online platform. As FGD participants felt uneasy reading online materials and interactions, more effort is required from facilitators to collect information and enliven the environment. Several other suggestions have also been identified through the FGDs, including (1) finding terms that are more familiar to the general public, (2) providing examples that are applicable and relevant to each stage of disease progression, and (3) providing more comprehensive materials regarding the bio-psycho-sociocultural and spiritual dimensions.

Additionally, the reading culture in Indonesia requires improvement. Indonesia ranks second-to-last in the world in terms of literacy, which indicates that reading interest among its population is extremely low, with only 0.001%, or one out of every thousand Indonesians, being avid readers. In addition, the Most Literate Nation in the World survey ranks Indonesia sixty-first out of sixty-one countries (31).

The iSupport materials and the use of electronic devices are challenging for older Indonesian people in the implementation process, especially for those with low education. Therefore, the use of iSupport was designed for participants with a minimum high school education to meet the minimum standard of understanding of the training materials. Finally, modules should also be divided into several sub-modules to allow the audience to be more focused.

Although, according to the inclusion criteria, the FGD participants had provided care for PwD for at least 2 years, not all caregivers experienced the various clinical stages of dementia care. This resulted in suboptimal contributions from participants on specific topics. Due to the pandemic, the translational adaptation phase of this study was conducted online, which may have reduced communication interactions between participants.

In conclusion, our study goes through 5 stages of the translation and adaptation approach process. Involve stakeholders related to dementia care. Adjustments in words, expressions, character names and the suitability of terms with local Indonesian culture are the main parts of this translation and adaptation process. The entire process has undergone expert panel appraisal and fidelity checks to ensure adaptation and translation compatibility with the original version of iSupport Dementia. The data obtained from the FGDs was very beneficial for the translation and adaptation process into the Bahasa Indonesia version. The translation and adaptation process of the WHO iSupport program still needs to be expanded to be more specific to the various phases of the disease courses, as well as local cultural experiences that the caregiver of people with dementia experience. Feedbacks related to content and choice of words are the central aspect that should be enhanced to ensure the adapted program is suitable for the local culture. We hope that the results of the translation adaptation of this module will serve as a benchmark for developing programs to assist caregivers in providing PwD care, thereby enhancing the quality of life for PwD and their caregivers.

## Data availability statement

The raw data supporting the conclusions of this article will be made available by the authors, without undue reservation.

## Ethics statement

This study has obtained ethical clearance with letter number: 14/02/KEP-FKIKUAJ/2021 from School of Medicine and Health Sciences Atma Jaya Catholic University of Indonesia. The patients/participants provided their written informed consent to participate in this study.

## Author contributions

YT and TN designed and directed the project. YT, KK, IS, TS, YH, KT, and TN drafted the manuscript. KK, IS, and YT aided in interpreting the results and worked on the manuscript. All authors discussed the results and commented on the manuscript.
